# Primary Lateral Sclerosis: An Overview

**DOI:** 10.3390/jcm13020578

**Published:** 2024-01-19

**Authors:** Veria Vacchiano, Luigi Bonan, Rocco Liguori, Giovanni Rizzo

**Affiliations:** 1 IRCCS, Istituto delle Scienze Neurologiche di Bologna, UOC Clinica Neurologica, 40139 Bologna, Italy; veria.vacchiano2@unibo.it (V.V.); rocco.liguori@unibo.it (R.L.); 2 Department of Biomedical and Neuromotor Sciences (DIBINEM), University of Bologna, 40126 Bologna, Italy; luigi.bonan@studio.unibo.it

**Keywords:** primary lateral sclerosis, hereditary spastic paraplegia, ALS, motor neuron disease, review

## Abstract

Primary lateral sclerosis (PLS) is a rare neurodegenerative disorder which causes the selective deterioration of the upper motor neurons (UMNs), sparing the lower motor neuron (LMN) system. The clinical course is defined by a progressive motor disability due to muscle spasticity which typically involves lower extremities and bulbar muscles. Although classically considered a sporadic disease, some familiar cases and possible causative genes have been reported. Despite it having been recognized as a rare but distinct entity, whether it actually represents an extreme end of the motor neuron diseases continuum is still an open issue. The main knowledge gap is the lack of specific biomarkers to improve the clinical diagnostic accuracy. Indeed, the diagnostic imprecision, together with some uncertainty about overlap with UMN-predominant ALS and Hereditary Spastic Paraplegia (HSP), has become an obstacle to the development of specific therapeutic trials. In this study, we provided a comprehensive analysis of the existing literature, including neuropathological, clinical, neuroimaging, and neurophysiological features of the disease, and highlighting the controversies still unsolved in the differential diagnoses and the current diagnostic criteria. We also discussed the current knowledge gaps still present in both diagnostic and therapeutic fields when approaching this rare condition.

## 1. Introduction

Primary lateral sclerosis (PLS) is a sporadic neurodegenerative disease characterized by the progressive degeneration of the upper motor neurons (UMNs). It is currently considered as an extreme end of the spectrum of motor neuron diseases (MNDs), of which amyotrophic lateral sclerosis (ALS) is the most represented condition. Although it may share, especially in the early phase, some clinical features overlapping with ALS, PLS is marked by the lack of clinical involvement of the lower motor neurons (LMNs), and by a more protracted clinical course with a better prognosis [1].

The disease was firstly described by Charcot in 1865, but, only ten years later, Erb reported a clinical phenotype characterized by the isolated involvement of the corticospinal tracts named “spastic spinal paralysis” [2].

The prevalence of PLS is estimated to be around 2–3% of total cases of MND [3]. However, this data strongly depended on the population considered, since other studies sustained a higher prevalence (up to 5% of the MND population [4]). The incidence is thought to be less than 0.1/100,000/year, even though the largest population-based study [5] describing the epidemiology of PLS in Catalonia in the period of 2011–2019 and in Valencia in the period of 2013–2019 pointed out an estimated incidence ranging from 0.2 to 0.6 per 100,000 people per year (higher than expected from previous data).

A male predominance has been consistently observed in PLS (range of 2–4:1) [6,7], although other studies suggested a higher prevalence among females [5,8,9]; no difference between races have been reported [8,9,10].

The clinical course is characterized by a progressive motor disability due to muscle spasticity which typically involves lower extremities and bulbar muscles.

Although PLS has been traditionally considered as a “pure” motor neuron disorder, some “extra-motor” features have been systematically recognized in recent years, and supported by post-mortem and imaging reports that have consistently demonstrated cortical and subcortical changes beyond the motor cortex and corticospinal tracts [11]. Extramotor involvement mainly includes some neuropsychological deficits, isolated or widespread, sometimes configuring a frank frontotemporal dementia, and extra-pyramidal features.

There is a general agreement about the fact that PLS is an extremely rare disease, and part of the scientific community still doubts its existence as a unique entity.

In the classical original study from Le Forestier et al. [12] on 20 patients with a diagnosis of PLS, the presence of mild, not-progressing, or even transient LMN signs at EMG, confirmed by muscle biopsy, together with findings from previous post-mortem studies [13,14], led to the consideration of PLS as one end of the continuous spectrum of motor neuron diseases, rather than as a discrete entity. However, the clinical imprecision in the diagnosis, together with some uncertainty about overlap with UMN-predominant ALS, has become an obstacle to the development of specific therapeutic trials. Furthermore, a further difficulty can be found in the differential diagnosis with hereditary spastic paraplegia (HSP).

In this narrative review, we provided a comprehensive analysis of the existing literature, including neuropathological, clinical, neuroimaging, and neurophysiological features of the disease, and highlighting the controversies still unsolved in the differential diagnoses and the current diagnostic criteria. We also underlined the current knowledge gaps still present in both diagnostic and therapeutic fields when approaching this rare condition.

## 2. Neuropathology, Neurobiology, and Genetics of PLS

The main histopathological features of PLS consist of diffuse brain atrophy, the loss of pyramidal neurons in the fifth layer of the precentral gyrus, the degeneration of the white matter associated with cortico-spinal tract, and the relative sparing of lower motor neurons. The degeneration of the primary motor cortex and pyramidal tract is always present, while other findings (such as the involvement of the prefrontal and temporal cortices and ubiquitinated inclusions) are inconstant; damage of the LMNs is quite rare and tend to be slight and isolated [15].

Up to the 1980s, lots of case reports were published reporting standard descriptions of the macro- and microscopic examination of the central nervous system of patients affected by PLS. For instance, Beal et al. [16] illustrated a case with severe atrophy of the precentral gyrus of the cerebral cortex bilaterally, accompanied by a general sparing of the remaining cortices and thinning of the pyramids of the medulla. One of the first and most important reviews about PLS was published by Pringle et al. [17], who described a picture characterized by a complete loss of UMNs in the fifth layer of the motor cortex associated with a chronic degeneration of the cortico-spinal tract; LMNs were found intact and there was no involvement of other cortical areas. In general, the first pathological studies published in literature agreed on the main features of this disease, reporting a condition associated with the loss of Betz cells in the precentral gyrus. Similar data have been confirmed by a subsequent study [18], in which the authors have applied an automated analysis program and confirmed a marked total brain atrophy associated with a focal atrophy of other structures, such as the corpus callosum (especially the mid-anterior, central, and mid-posterior parts, which encompass sensory-motor fibers and projections from the dorsal–prefrontal and superior frontal cortices) and thalami (via a mechanism of Wallerian degeneration), finding a correlation between the atrophy degree and clinical severity [18].

In the late 1990 to early 2000s, several immunohistochemical studies have been published reaching different results. Some of them proved the accumulation of ubiquitin not only in the motor cortex, but also in the prefrontal and temporal cortex, depicting a condition similar to fronto-temporal lobar degeneration and excluding the presence of Bunina bodies, which were thought to be more specific of ALS than other MNDs [19]. Other authors confirmed the presence of ubiquitin-positive inclusions associated with dystrophic neuritis in layer II of the frontal and temporal cortex, accounting also for the presence of isolated Bunina bodies and neuronal inclusions in the posterior part of the putamen and in the lower motor neurons [14,15]. A single case report also described a dendritic ballooning phenomenon, which appeared to be very rare in this condition [20].

The crucial role of the transactive response DNA-binding protein of 43 kDa (TDP-43) in the major part of the MND pathology was first reported by Neumann and colleagues in 2006 [21]. They demonstrated that ubiquitinated cytoplasmic inclusion bodies—already known from the previous descriptions of PLS and FTD pathology—also contained the aggregation of TDP-43. They demonstrated a translocation of TDP-43 from the nuclei to cytoplasm (in association with ubiquitin inclusions) in the cells affected by the disease. This evidence strengthened the already existing idea that MND and fronto-temporal lobar degeneration had been part of a spectrum [22,23]. In line with this evidence, Kosaka et al. [24] reported a new case and re-examined the case originally reported by Tan et al. [15], demonstrating prominent frontotemporal atrophy, and abundant TDP-43 pathology throughout the cerebral neocortex and hippocampus, but only a few inclusions in LMNs, which were substantially preserved. Furthermore, Hirsch-Reinshagen et al. [25] described a family with a TBK1 genetic variant, in which two siblings presented with a combination of PLS and primary progressive aphasia. In both, TDP-43 pathology was present throughout the neocortex, limbic cortex, and many subcortical regions; however, the LMNs were well-preserved and only a single TDP-43 cytoplasmic inclusion was detected in the lumbar spinal cord in one of the two cases.

MacKenzie et al. recently reported the neuropathological findings in seven cases of PLS, revealing the presence of TDP-43 inclusions mainly localized in the primary motor cortex and cortico-spinal tract, but also in the LMNs, although sparse and not associated with substantial pathological changes [26]. The authors confirmed the TDP43 pathology is shared by PLS and ALS, but PLS possess some protective factors against LMN degeneration.

However, the limitations in these studies are the selection of patients mainly classified on the basis of their pathological findings, but with insufficient clinical information to determine if they actually fulfilled the clinical PLS criteria. On the other hand, a number of neuropathological reports demonstrated that PLS can be a rare clinical phenotype of other neurodegenerative diseases, such as Alzheimer’s disease and Lewy body disease [27], progressive supranuclear palsy [28], neuronal intermediate filament inclusion disease [29], globular glial tauopathy [30], and argyrophilic grain disease [31].

The precise molecular and cellular mechanisms of UMN degeneration in PLS remain mostly unknown. Numerous related cellular defects may result in UMN vulnerability, as demonstrated through several mouse models generated based on PLS-linked genetic variants. One of the recognized mechanisms underlying UMN vulnerability is based on intracellular trafficking defects. Indeed, corticospinal motor neurons are selectively vulnerable to the lack of expression of Alsin, a large protein encoded by the *ALS2* gene, implicated in a wide range of cellular functions ranging from endocytosis, membrane trafficking, endolysosomal protein degradation, and apoptotic signaling from mitochondria upon cellular stress [32]. In mouse models, the depletion of Alsin caused the disintegration of corticospinal motor neurons at several levels (cervical spinal cord, pyramidal decussation, and pons) and the disruption of apical dendrites with numerous vacuoles, as well as profound defects in the morphology and function of the mitochondria and the Golgi apparatus [33].

Significant ultra-structural defects in the Golgi apparatus and mitochondria suggest problems with ATP production and energy metabolism, as well as the post-translational modification of proteins and lipid homeostasis [33]. Indeed, PLS-patient-derived fibroblasts have shown elevated ATP demand and consumption, thus needing an enhanced energy metabolism through both oxidative and glycolytic ATP pathways, which, in turn, led to an overproduction of reactive oxygen species [32].

Interestingly, recessive loss-of-function mutations in the *ALS2* gene have been identified in atypical forms of PLS with infantile or juvenile onset [34,35,36], infantile ascending spastic paraparesis [37], and hereditary spastic paresis [35,38], with overlapping phenotypes and no clear genotype–phenotype correlation.

Actually, although the current consensus criteria [7] state that the “screening of panels for pathogenic genetic variants associated with spastic paraparesis should be performed only in cases of progressive UMN syndromes restricted to symmetrical lower limb involvement”, our current knowledge of HSP and ALS genetics is widely incomplete. In fact, some families with multiple members affected by PLS have been reported [39,40,41,42], and, for this reason, in the current criteria, there is not mention of “lack of family history”, which was, instead, considered as a clinical criterium in the previous set of criteria (Pringle) [17].

One of the largest studies [43] which analyzed the *C9orf72* gene in a PLS cohort identified the presence of the expansion in 1 patient out of 110. Another relatively large study found that 18% of patients carried a variant in either ALS (*C9orf72*), Parkinson’s disease (*PARK2*), or HSP (*SPG7*) genes [44]. A predicted pathogenic mutation in the *SYNE2* gene was also identified [44].

Among HSP-related genes, *SPG7* variants have been linked to a PLS-like presentation in several studies [45,46], while, among rarer ALS-associated genes, *TBK1* genetic variants [47] have been reported in a family with PLS. Furthermore, *FIG4* [48], *UBQLN2* [49,50], and *OPTN* variants [51] have been associated with UMN-predominant MND phenotypes resembling PLS. Besides *ALS2* [34,35,36], juvenile primary lateral sclerosis (JPLS) has also been linked to *ERLIN2* [52] variants.

**Figure 1:**
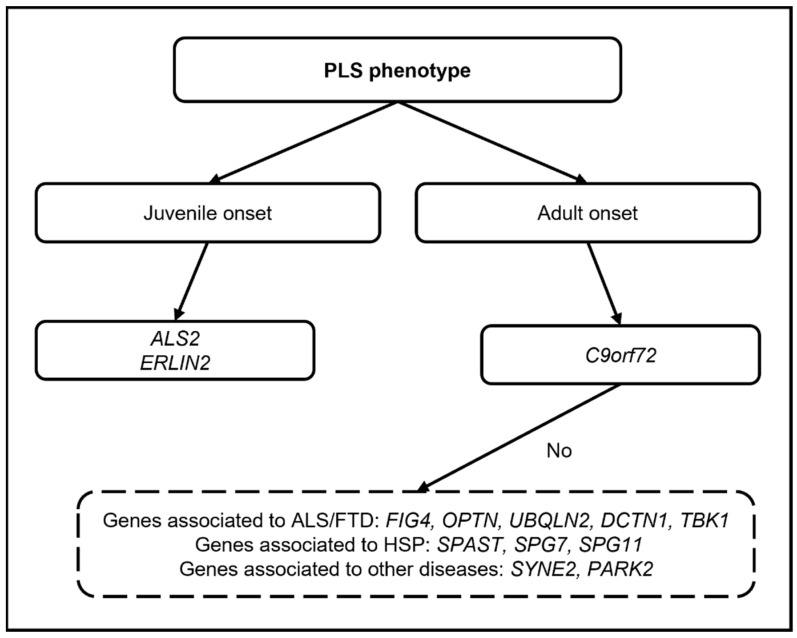
Diagram showing the possible genetic work-up in PLS (only genes for which pathogenic variants have been reported are mentioned).

In the most recent and largest genetic study on 139 PLS patients [53], likely pathogenic or pathogenic variants in genes related to ALS-FTD (*C9Orf72*; *TBK1*), HSP (*SPAST*; *SPG7*), and the ALS-HSP-Charcot-Marie-Tooth overlap (*NEFL*; *SPG11*) were found in 7% of the cohort, remarking upon the possible significant contribution of genetics in the diagnostic work-up of PLS (Figure 1).

## 3. Clinical Features

The mean age of clinical onset in PLS is around 50 years, which is about a decade earlier than non-familial ALS, and a decade later than HSP. In the most part of the cases (90%), the onset of symptoms insidiously involves the lower limbs, and patients may complain of a “loss of fluidity” and/or a “loss of stability” in the gait. However, for a significant minority of the patients, a bulbar onset has been described, including dysarthria, nasal speech, and emotional lability configuring a pseudobulbar affect [54]. Dysphagia can be present but usually is not that severe so as to require gastrostomy as in ALS. Similarly, the need for ventilatory support is quite exceptional. In fact, in the prospective NEALS PLS registry [54] of 250 PLS patients, with a three-year median follow-up after enrollment, only 7% required a feeding tube and less than 1% needed permanent assisted ventilation. Usually, PLS slowly generalizes to the upper limbs, while a focal onset involving an upper limb is extremely rare [55]. The rate of progression is much slower than typically encountered in ALS, with an average disease duration ranging from 7.2 to 14.5 years [6].

Depending on the patient’s age and comorbidities, the prognosis of PLS is at least a decade from the onset of symptoms and often significantly longer [54].

Typically, the neurological examination shows only upper motor neuron signs, including spasticity and the spread of reflexes, with the absence of lower motor neuron findings (fasciculations and muscle wasting). Stiffness as a presenting symptom is observed more commonly in PLS than ALS (47% vs. 4%), and limb wasting is rare in PLS (~2%) [56].

An upper motor neuron pattern of weakness may be observed (extensors in upper extremity; flexors in lower extremity), but symptoms referred by the patients are often a combination of increased tone, decreased co-ordination, and mild weakness.

Although the involvement of the lower limbs in PLS is commonly symmetrical, a progressive hemiplegia is a very rare phenotype originally described eponymously by Mills [57]. This latter condition, also known as the “hemiplegic variant”, is characterized by slow progressive ascending weakness, usually starting in a distal lower limb, and then progressing to a proximal ipsilateral lower limb and upper limb, associated with pyramidal signs on the affected side, and sometimes also on the contralateral one [58]. Facial and bulbar weakness may be present, as well as slight sensory disturbances [59]. In most patients, the syndrome remained strictly unilateral after 15 years, although the involvement of the contralateral side has been reported in about 30% [59]. The scarcity of reports on this condition, as well as the paucity of complementary resources necessary to better define its pathophysiological mechanisms, led to doubt about the authenticity of this entity [58]. Sensory disturbances or deficits should not be observed in PLS. Among additional clinical features, the most consistently reported are bladder instability with urinary frequency and retention [60], extrapyramidal features [61,62], and cognitive disorders [62,63,64,65,66,67].

The most common neuropsychological deficits in PLS include problems in social cognition, apathy, executive dysfunction, language, and verbal fluency [62,63,64,65,66,67]. Furthermore, the co-presence of full-blown frontotemporal dementia, which was thought to be relatively rare (2%) [67], was recently found to be more common than expected in PLS patients [62].

Abnormalities in ocular movements, especially the loss of smooth pursuit, and even supranuclear palsy [17], may be present, and saccadometry has shown the loss of fixation and, particularly, prominent antisaccade errors compared to ALS patients [68].

Clinical disability in PLS is evaluated by clinical examination, but combined UMN scores and scales developed for other MNDs are also commonly used. These scales include the revised ALS Functional Rating Scale (ALSFRS-r) [69], the Penn Upper Motor Neuron Score (PUMNS) [70], the Modified Ashworth Scale [71], the emotional lability questionnaire [72], and the more recently validated PLS functional rating scale (PLSFRS) [73].

## 4. Diagnostic Criteria

Over the years, different sets of criteria were proposed to diagnose PLS.

**Figure 2:**
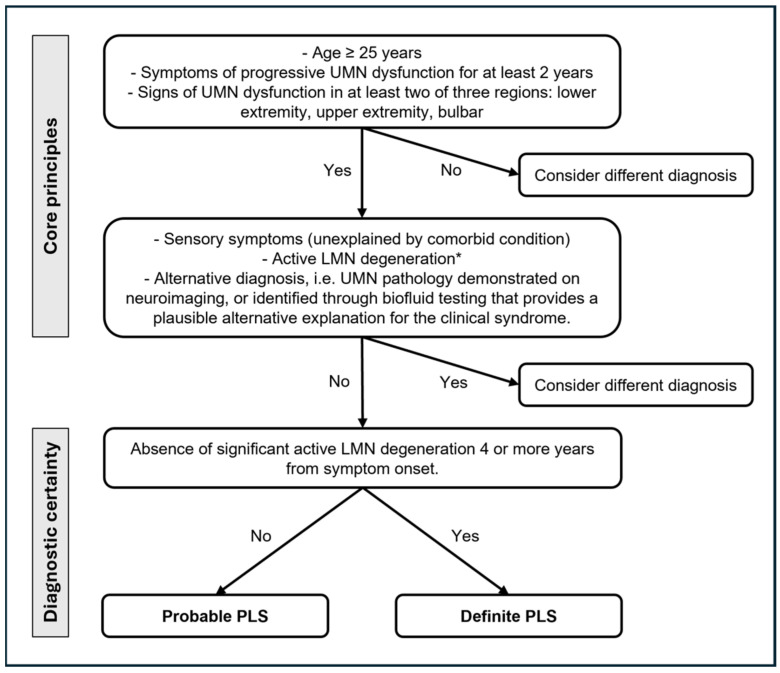
Current diagnostic criteria for PLS [7]. UMN: upper motor neuron; LMN: lower motor neuron. * Minimally increased insertional activity and positive sharp waves or fibrillation potentials in extremity muscles are allowed.

In 1945, the PLS diagnostic criteria [74] suggested a minimum of a five-year symptom duration for diagnosing PLS, while, in 1992, the Pringle criteria [17] proposed that a minimum symptom duration of three years would have permitted a reliable diagnosis, still describing as core features an adult onset of insidious spastic paresis in the lower limbs (but also in the bulbar or upper extremities), usually symmetric and in the absence of a family history. In 2006, the Gordon criteria [75] recommended a symptom duration of four years to label the diagnosis. Finally, the recent 2020 consensus diagnostic criteria (Figure 2) [7], recognizing the implications of diagnostic delay, introduced a category of “probable PLS” for patients with isolated UMN symptoms in at least two of three regions (lower extremity, upper extremity, and bulbar) for 2–4 years. The recognition of a pragmatic category of “probable PLS” reflects the desire to facilitate the earlier inclusion of patients with PLS in future trials of potentially disease-modifying therapy before the disability becomes advanced.

## 5. Neurophysiological Features

The main diagnostic challenge remains the discrimination of PLS from UMN-predominant ALS patients, especially in the early phase of the disease, when the borderline between these two entities is difficult to delineate. This issue is complicated by the evidence of minimal and not-progressive electromyographic (EMG) denervation signs in some PLS patients [10,17,76,77].

In a study on 29 patients with pure UMN involvement at the initial visit, 13 were later classified as UMN-predominant ALS on average between three and four years from the onset of symptoms, due to the development of denervation, chronic motor unit changes, and fasciculation potentials in one to two muscles at EMG, as well as limited clinical LMN signs. Four of these patients eventually met the WFN El Escorial clinical trial criteria for ALS [75].

In another study on 25 PLS patients, the authors observed a more aggressive and faster disease in patients with evidence of active denervation potentials (increased insertional activity, fibrillations, and/or positive sharp waves) in one or more muscles, even though they did not meet the neurophysiological criteria for ALS [10].

In a large multicentric cohort of 217 patients with pure UMN disease, subjects were categorized into two groups according to the presence or absence of minor denervation signs. The authors found no differences between the two groups in terms of the site of onset, frequency of clinical symptoms, ALSFRS-R scale, vital capacity, or use of non-invasive positive pressure ventilation [77], suggesting that subtle EMG abnormalities can not necessarily be used as a prognostic tool in patients with clinical UMN disease.

A more recent study [78] confirmed these findings of minimal denervation activity in single muscles of PLS patients without a clear progression, even though the authors observed a faster disease progression in patients with a greater amount of EMG abnormalities.

These findings were subsequently corroborated by another cohort study [79] where 21 patients with PLS syndrome associated with definite but limited EMG denervation changes were followed up with for a median of seven years, and around 90% of this cohort maintained the PLS phenotype and diagnosis.

To conclude, although PLS patients lack evident clinical lower motor neuron signs on the neurological examination, several studies report minor and stable changes with needle EMG, including sparse fibrillations, fasciculations, and enlarged motor unit potentials, generally limited to one or two muscles [10,17,76,77]. After four years, the probability of developing new lower motor neuron findings on the EMG becomes low (~20%) [75].

For this reason, EMG findings consistent with mild and not-progressing involvement of lower motor neurons are tolerated in the category of “probable PLS”, coined in the last set of diagnostic criteria [7].

Conversely, if a patient has EMG denervation and, subsequently, developed focal LMN signs and symptoms over the course of four years, but still does not meet the criteria for ALS [80], a diagnosis of UMN-predominant ALS would be more appropriate than PLS [7]. However, the reason for this resistance to LMN degeneration in PLS, at variance from ALS, is widely unknown. The most obvious explanation is that PLS and ALS syndromes present different underlying pathogenic processes.

Besides the LMN assessment, neurophysiological tools can be used to quantify UMN involvement. The most conventional tool is transcranial magnetic stimulation, which have proven abnormalities in the motor-evoked potentials, showing the absence of reproducible cortical responses or longer central motor conduction times in PLS compared to ALS [76]. Furthermore, high threshold measures for cortical stimulation, which suggest cortical inexcitability, are a specific signature of PLS, probably reflecting a greater degree of neurodegeneration within the motor cortex and the corticospinal tracts, as the resting motor threshold reflects the density of corticomotoneuronal projections into spinal motor neurons, as well as the excitability of large motor cortical neurons. These findings also reliably distinguish PLS from HSP [81], where cortical excitability is preserved.

## 6. Neuroimaging

Conventional imaging studies in PLS are primarily used to rule out alternative causes of isolated UMN dysfunction at the brain or spinal cord levels.

However, with the advancement of both structural and functional imaging technologies over the years, several studies attempted to examine deeper aspects of the pathogenesis and to define specific signatures of PLS [82].

**Figure 3:**
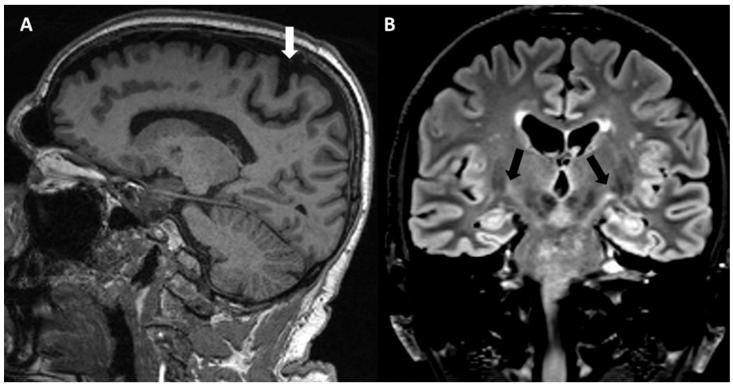
MRI in a PLS patient. (**A**) Sagittal T1 sequence showing atrophy of primary motor cortex with enlargement of central sulcus (white arrow). (**B**) Coronal flair sequence showing bilateral corticospinal tract hyperintensities (black arrows).

Obviously, the main brain structure altered and investigated in PLS is the pyramidal pathway. Case reports and case series reported some qualitative abnormalities in conventional magnetic resonance imaging (MRI) (Figure 3), such as the focal atrophy of the precentral gyrus, sometimes with a “knife edge” appearance of the gyri [83]. Furthermore, corticospinal tract (CST) hyperintensities in the brain and spinal cord [84,85] have been reported on T2- and FLAIR-weighted images, occasionally with a “wine glass” appearance in the coronal view at the diencephalic level [86], and the hypointensity of primary motor cortex (motor band sign) on susceptibility-weighted imaging (SWI) [87]. However, these changes are not specific to PLS, and have been systematically reported in ALS [4,88], and also in HSP, although with an apparently lower frequency [88].

Cross-sectional quantitative imaging studies invariably confirmed the atrophy of the precentral gyrus [18,89], the reduction of the premotor cortex surface area [76], as well as its focal thinning [90,91,92], while the CST pathology was highlighted by the increase in diffusivity and reduction of fractional anisotropy by means of diffusion tensor imaging (DTI) studies [93,94,95]. The motor pathway in PLS was also evaluated by metabolic and functional techniques. Proton MRI spectroscopy studies of the premotor cortex in PLS patients showed reduced N-acetyl aspartate (NAA)/creatine (Cr) ratios [55,96,97] and increased myo-inositol/Cr ratios [98], consistent with neuronal dysfunction or loss, and gliosis, respectively. Functional MRI studies reported increased functional connectivity in PLS [99,100], which were interpreted as an adaptive, compensatory process, similarly to ALS [101]. A few [18F]-FDG PET studies in PLS have been published, but failed to find a “signature” of PLS as compared to ALS, mainly showing a prominent hypometabolism of the prefrontal and premotor cortices [102]. However, recent advances in metabolic imaging suggest that a combined cord and brain [18F]-FDG PET may differentiate ALS from PLS [103]. Several ligand PET studies have been conducted with exploratory purposes, but they are beside the scopes of this review.

Extra-motor cortical and subcortical involvement in PLS has been variably reported by volumetric, vertex, and morphometric analyses, ranging from pathology in specific brain regions, including the thalamus, caudate, and hippocampus [104,105,106], to widespread parietal, prefrontal, cerebellar, and brainstem degeneration [107,108,109]. Certain structures such as the thalamic motor and sensory nuclei seem to be selectively affected in PLS [105,106]. Over 20 diffusion tensor imaging studies, other than confirming the CST pathology [93,94,95], revealed corpus callosum [98,110] and cerebellar [107,111] abnormalities. Interestingly, a recent study [107] on 42 patients diagnosed with PLS assessed by volumetric and voxelwise analyses revealed several focal cerebellar alterations along with significant diffusivity alterations within the superior cerebellar peduncle, indicating the disruption of the main cerebellar outflow tracts. These findings supported a cerebro-cerebellar connectivity disruption which probably contributes to the motor disability in PLS. Other DTI studies have specifically highlighted extra-corpus callosum diffusivity alterations involving the superior and inferior longitudinal fasciculi, fornix, thalamic radiations, and parietal lobes [112,113]. At variance from ALS, there is a relative lack of longitudinal imaging studies in PLS. Existing longitudinal studies in PLS are limited due to cohort size limitations, typically presenting only two timepoints, and varying considerably in follow-up intervals [97,114,115,116]. Interestingly, a study of eight suspected PLS patients who initially did not fulfill the diagnostic criteria exhibited progressive precentral gyrus thinning and increasing functional connectivity [117]. Other studies of suspected PLS patients showed connectivity and gray/white matter abnormalities even before meeting the diagnostic criteria [117,118]. A more recent study on 41 PLS patients, through a 3D T1-weighted structural, diffusion tensor imaging, and resting-state functional MRI protocol, confirmed the progressive primary motor cortex degeneration, the significant supplementary motor and pre-motor area involvement, the progressive brainstem atrophy, the cortico-medullary and inter-hemispheric disconnection, and the close associations between clinical upper motor neuron scores and somatotopic connectivity indices in PLS, highlighting that PLS should not be considered as a “benign” motor neuron disease [119].

## 7. Differential Diagnoses

As previously stated, the differential diagnosis for PLS might be challenging because there are many different conditions which can mimic its clinical features.

The most represented disease which clinically overlaps with PLS is hereditary spastic paraparesis (HSP), a genetic syndrome characterized by progressive weakness and spasticity in the lower limbs, caused by around 70 genetic variants recognized so far, with all possible patterns of inheritance reported [120]. The age of onset can be variable, but, usually, the more common autosomal-dominant forms occur between the second and third decades. HSP is historically classified in “pure”, if the spastic paraplegia together with the subtle involvement of the dorsal column are the primary manifestations, and “complicated” if other additional features are present, encompassing dementia, cognitive delay, epilepsy, neuropathy, and others [120]. In the most common form of autosomal-dominant disease, the SPG4 (caused by genetic variants in the gene encoding for spastin) is associated with a “pure” phenotype, while, among the autosomal-recessive forms, SPG11 is the most frequent, and associated with a “complicated” phenotype.

Some clinical elements may help in distinguishing HSP from PLS, such as the presence of a family history (PLS is mainly considered a sporadic disease), the earlier and symmetric onset of the disease, the presence of a diminished vibratory sensation on clinical examination, the absence of bulbar involvement, and the slower disease progression in patients with HSP compared to PLS [121]. Nonetheless, the clinical distinction between these two entities could be practically impossible in some circumstances, and genetic testing remains essential for ruling out HSP as the etiology for an apparently sporadic adult-onset UMN syndrome with leg onset.

This is especially true when PLS started with lower extremity involvement [122], or in some specific forms of HSP (i.e., spastic paraplegia types 4 and 7) where the clinical onset could be in later adulthood [123]. Furthermore, asymptomatic UMN signs or a frank clinical involvement of the upper limbs may be observed in HSP cases [124], as well as asymmetry [125]. Moreover, a negative family history can occur in 40% of cases due to recessive or X-linked inheritance, or even de novo genetic variants [125].

To further complicate this issue, a study on 90 patients with apparently sporadic UMN syndrome, categorized in phenotypes of HSP (involvement of legs only), HSP-PLS overlap (involvement of arms and legs), and PLS (bulbar involvement) showed significant overlap in the age of symptom onset and no differences between the groups in features classically used to distinguish the two diseases, such as mild dorsal column dysfunction (decreased vibratory sense or abnormal leg somatosensory evoked potentials), symptoms of urinary urgency, or mild electromyographic abnormalities [126].

Among neurodegenerative diseases, as previously reported, the UMN-predominant ALS is the most difficult differential diagnosis. This entity is defined by the presence of motor disability mainly secondary to UMN signs with known EMG and/or clinical LMN signs that do not meet the criteria for clinically definite, clinically probable, or probable-laboratory-supported ALS as defined by the revised El Escorial criteria [80,127]. Interestingly, the progression of patients with UMN-predominant ALS is slower than typical ALS patients [128], probably suggesting that this phenotype lies in the area between PLS and ALS along the complex spectrum of the disease. However, an interesting study [127] showed that patients with isolated UMN signs at the first visit, but destined to develop LMNs in the next four years, presented, more frequently, a bulbar onset and a shorter time to the first evaluation (an indirect sign of the disease progression rate) than the PLS group. Additional findings of muscular atrophy on initial examination, weight loss, and any MRC grade less than 4 at the initial visit were predictive of UMN-predominant or classic ALS as compared to patients remaining with a PLS phenotype. Interestingly, patients with PLS may develop weakness, but it was usually mild and often generalized in a UMN distribution.

Finally, reduced FVC was related to UMN-predominant or classic ALS groups, suggesting that FVC was a measure of LMN function in the phrenic nerve more than of UMN involvement.

Other mimics of PLS can be subdivided into: metabolic disorders (adrenomyeloneuropathy); neuroinflammatory diseases (e.g., primary progressive multiple sclerosis, and anti-amphiphysin paraneoplastic syndrome); and infections such as human T-cell leukemia virus type 1 and 2 (HTLV1 and HTLV2 myelopathy) and neurosyphilis. Other brain and spine structural abnormalities are relatively easily “ruled out” by brain and spine imaging, such as multiple infarcts, cervical spondylosis, syringomyelia, Chiari malformation, compressive foramen magnum lesions, and spinal cord tumors.

Among inflammatory diseases, progressive solitary sclerosis [129] deserves a special mention. This is a rare entity which may present with both symmetrical and unilateral UMN features of progressive motor impairment, but it is considered a localized variant of multiple sclerosis, due to the presence of a single demyelinating lesion in the central nervous system and of cerebrospinal fluid (CSF) oligoclonal bands.

The differential diagnoses of PLS are summarized in Table 1.

## 8. Biofluid Biomarkers

Biomarkers are an area of active interest to exploit CSF or blood components which may add a diagnostic and/or prognostic value in the assessment of neurodegenerative diseases.

Among biofluid biomarkers, neurofilaments have emerged as promising diagnostic and prognostic biomarkers in motor neuron diseases [130]. Several studies have shown that both the neurofilament light chain (NfL) and phosphorylated neurofilament heavy chain (pNfH) are highly elevated in ALS and correlate with measures of disease progression [131,132,133].

Some studies have shown lower levels of NfL in PLS compared to ALS [134,135], reflecting its much slower progression, although other studies have not shown significant changes between the two groups [136,137], probably due to the small number of PLS patients included. Interestingly, in some cohorts, NfL was found to be lower in PLS than in UMN-predominant ALS patients, with practical diagnostic relevance, given the relatively better prognosis of PLS [138,139].

Additionally, cerebrospinal fluid chitinases, thought to be macrophage-derived, were found to be lower in PLS compared to ALS [140,141], and showed the best performance in distinguishing these two categories [141], reflecting a lesser extent of microglial neuroinflammation in PLS, which, in turn, may be linked to axonal loss. However, these observations deserve to be confirmed in multicentric larger cohorts, and further studies are needed to explore the key distinction of PLS from UMN-predominant ALS.

## 9. Management and Treatment

Currently, there are no disease-modifying treatments approved or tested in experimental trials for PLS, probably due to the rarity of the disease and lack of significant understanding of its underlying pathophysiology. Therefore, the approach to treatment remains essentially targeted to alleviate the symptoms and improve the quality of life of patients. Since clinical trials of symptomatic treatments are limited, clinical experience based on other neurological disorders with similar symptoms has been used to guide treatments. A multidisciplinary approach should be preferred in order to manage several disturbances that can be present along the disease course, such as spasticity, fatigue, pseudobulbar palsy, pain, depression, and sphincteric and sexual dysfunctions. Non-medication approaches including physical and occupational treatment are essential for gait and balance training, the reduction of the discomfort from muscle stiffness, and evaluation for assistive devices, along with speech therapy, pneumological assessment, and psychosocial support. Experimental data on physiotherapy in patients with PLS are lacking, but a strictly monitored exercise program has been proposed to potentially reduce motor deterioration in patients with ALS [142]. Preliminary observations suggest that combining robot-aided and conventional rehabilitation could be a promising approach to mitigate the PLS disability burden [143].

For spasticity, which is the most disabling symptom, the first-line oral agents include baclofen, tizanidine, benzodiazepines (e.g., clonazepam and diazepam), gabapentin, pregabalin, and dantrolene. These drugs can cause some side-effects, most commonly somnolence and the worsening of hyposthenia. For patients who achieve some benefit with anti-spasticity drugs, but are limited by these side-effects, a trial of intrathecal baclofen—and subsequent baclofen pump placement—may be useful. In recent years, cannabinoids have been increasingly used to treat spasticity with significant improvement and without serious adverse events [144], and delta-9-tetrahydrocannabinol and cannabidiol (THC/CBD: 50:50) spray (nabixomols) had a positive effect on spasticity symptoms in a placebo-controlled randomized phase 2 trial in patients with motor neuron disease [145]. Treatment with botulinum toxin type A associated with physiotherapy has also proven beneficial in the short term and long term in patients with moderate-to-severe spasticity [146,147]. Dalfampridine (4-aminopyridine) has also been shown to be effective in spasticity [148]. Furthermore, there are data to support the effectiveness of some non-pharmacological therapies such as transcutaneous electrical nerve stimulation [149] and acupuncture [150] on spasticity, although more controlled studies are needed.

The management of excess oral secretions or drooling is similar to that used in ALS [151]. Most patients have beneficial effects with oral anticholinergic medications—amitriptyline, scopolamine, glycopyrrolate, or atropine drops. For drooling unresponsive to oral therapies, botulism toxin injections into submandibular glands may be beneficial.

For pseudobulbar affect, selective serotonin reuptake inhibitors can help patients with emotionality even in the absence of depression [152,153,154]. Furthermore, the combination of dextromethorphan and quinidine (Neudexta) [155] may prove beneficial. Tricyclic antidepressants may be useful in patients without significant beneficial effects from Neudexta. Urinary urgency can be relieved by drugs such as oxybutynin.

## 10. Conclusions

PLS appears to be a rare but distinct disease, which, first and foremost, represents a diagnostic challenge, due to the clinical overlap with HSP and UMN-predominant ALS, especially in the early phases. The main knowledge gap in the field of clinical diagnosis is characterized by the lack of specific biomarkers that may contribute to improving our diagnostic accuracy. This is further complicated by evidence suggesting that the PLS phenotype may be the clinical expression of different neuropathological entities.

As stated before, PLS has been reported to be the predominant clinical phenotype in cases with a confirmed neuropathological diagnosis of dementia and atypical parkinsonism (including progressive supranuclear palsy, neuronal intermediate filament inclusion disease, globular glial tauopathy, or argyrophilic grain disease) [27,28,29,30,31]. PLS has also been described as a rare clinical phenotype of a variety of systemic diseases, including several conditions consisting of autoimmune [156] or metabolic error disorders [157]. However, these publications illustrated heterogeneous cases and might contain a bias towards unusual cases.

Despite the efforts of clinicians in implementing several sets of diagnostic criteria, currently, there is no gold standard for the diagnosis, which is necessarily based on recognizing characteristic clinical features and ruling out other potential causes.

As a result, the efforts in researching specific biofluid or imaging biomarkers are marred by diagnostic imprecision, which obviously creates bias in the selection of clinical cohorts. The addition of a histopathological gold standard to the diagnostic criteria for PLS would constitute a substantial step forward and should be focused on in future efforts.

Another promising field is the advancement of genetic testing, which might help to clarify the contribution of genetics to PLS susceptibility, as already shown by the association of rare genetic variants, classically responsible for other diseases, with the PLS phenotype. Thus, understanding the underlying pathophysiology of PLS would potentially guide therapy development, such as antisense-based approaches, as has already happened for other neurodegenerative diseases.

However, as is already the case for ALS, DNA testing may yield results that are difficult to interpret, thereby further complicating the clinical counseling of those patients who are already dealing with unsolved clinical questions.

From a clinical perspective, patients are worried about their prognosis and future; therefore, clinical research should focus on better defining the natural history of the disease, which cannot arise without an diagnostic advancement.

The most accurate diagnosis possible will, in turn, allow us to study more homogeneous patient samples with a view to obtaining a better pathophysiological interpretation of the disease and, last but not least, to identify possible disease-modifying treatments.

## Figures and Tables

**Table 1:** Differential diagnoses of primary lateral sclerosis.

Disease	Etiology	Clinical Hallmarks
Hereditary spastic paraparesis (most commonly type 4 or 7 for late-onset)	Genetic disorders	Symmetric paresis usually limited to lower limbs Slower progression Presence of family history or genetic variant
UMN-predominant ALS	Degenerative disorders	Faster progression Progressive development of clinical LMN involvement
Adrenomyeloneuropathy	Metabolic disorders	Impaired sensory vibration Elevated blood levels of adrenocorticotropic hormone Elevated serum levels of very long chain fatty acids Cerebral MRI white matter abnormalities Pathogenic variants in *ABCD1* gene
Primary progressive multiple sclerosis	Neuroinflammatory disorders	Presence of other neurological deficits (cerebellar dysfunction, brainstem syndromes, and visual loss) Demyelinating lesions of brain and cord Possible CSF oligoclonal bands
Progressive solitary sclerosis		Single demyelinating lesion in CNS Possible CSF oligoclonal bands
Anti-amphiphysin syndrome		Limbic encephalitis Dysautonomia Cerebellar dysfunction Positive anti-amphiphysin antibodies Presence of tumour
Tropical spastic paraparesis (HTLV-1 and -2 myelopathy)	Infectious diseases	Sphincter dysfunction Sensitive dysfunctions Positive serology
Neurosyphilis		Positive VDRL and TPHA Multisystemic involvement
Vascular and ischemic lesions	Brain and spine structural abnormalities	Imaging findings
Cervical spondylosis		
Syringomyelia		
Chiari malformation		
Compressive foramen magnum lesions		
Spinal cord tumours		

Abbreviations: CNS, central nervous system; CSF, cerebrospinal fluid; LMN, lower motor neuron; UMN, upper motor neuron; VDRL, venereal disease research laboratory; TPHA, treponema pallidum hemagglutination test.

## Data Availability

Not applicable.
